# Theoretical analysis of effects of transcranial magneto-acoustical stimulation on neuronal spike-frequency adaptation

**DOI:** 10.1186/s12868-022-00709-9

**Published:** 2022-05-02

**Authors:** Song Zhao, Dan Liu, Minzhuang Liu, Xiaoyuan Luo, Yi Yuan

**Affiliations:** 1 Institute of Integrative Medicine, Hebei University of Chinese Medicine, Shijiazhuang, China; 2 Department of Medical Imaging, The Second Hospital of Hebei Medical University, Shijiazhuang, China; 3 Institute of Electrical Engineering, Yanshan University, Qinhuangdao, China

**Keywords:** Spike-frequency adaptation, TMAS, Ermentrout model

## Abstract

**Background:**

Transcranial magneto-acoustical stimulation (TMAS) is a noninvasive technique that has advantages in spatial resolution and penetration depth. It changes the firing properties of neurons through the current generated by focused ultrasound and a static magnetic field. Spike-frequency adaptation is an important dynamic characteristic of neural information processing.

**Methods:**

To address the effects of TMAS on neural spike-frequency adaptation, this study employs some ultrasound and magnetic field parameters, such as magnetic flux density, ultrasonic intensity, fundamental ultrasonic frequency, modulation frequency, and duty cycle. Using these different ultrasound and magnetic field parameters, membrane potential curves, spike-frequency curves, and adapted onset spike-frequency curves are exhibited and analyzed.

**Results:**

The results show that spike-frequency adaptation is strongly dependent on ultrasonic intensity and magnetic flux density and is rarely affected by other parameters. However, modulation frequency and duty cycle influence membrane potentials and spike frequencies to some degree.

**Conclusions:**

This study reveals the mechanism of the effects of TMAS on neural spike-frequency adaptation and serves as theoretical guidance for TMAS experiments.

## Background

Transcranial magneto-acoustical stimulation (TMAS) is a new brain stimulation technique that has been studied in the neuroscience field. TMAS noninvasively stimulates neurons by generating an electric current with ultrasonic waves and a static magnetic field [1–5]. TMAS has more advantages in spatial resolution and penetrability than transcranial direct current stimulation and repetitive transcranial magnetic stimulation, which have been used in neuropsychiatric disease therapy [6, 7]. The high spatial resolution of TMAS benefits from its small ultrasonic spot, which is approximately 2 mm in diameter [8]. Transcranial focused ultrasound stimulation (TFUS) is another noninvasive brain stimulation technique that has been demonstrated to have spatial resolution and penetration equally as good as that of TMAS [9, 10]. However, our previous studies have shown that TMAS improves the neuromodulation performance in vivo animal experiments compared with TFUS [11, 12].

Spike-frequency adaptation is a significant neurobiological phenomenon in neural information processing, in which the spike frequencies of neurons gradually decline under constant external stimulation [13–15]. This effect can be attributed to two common types of adaptation currents, voltage-sensitive potassium currents (M-type currents, *I*_*M*_) and calcium-sensitive afterhyperpolarization currents (AHP-type currents, *I*_*AHP*_), which have different properties. Specifically, the M-type ones are active even in the quiescent state, while the AHP-type ones are activated by firing activity. Both types affect the dynamics of neurons, which deserves to discuss [13, 16–21]. Previous studies examined spike-frequency adaptation. For example, Benda et al. found that fast spike-frequency adaptation can result in intensity invariance of neural responses [16]. They further studied cases in which spike-frequency adaptation is suitable to be modeled as an adaptation current or a dynamic firing threshold, respectively [17]. Yi et al. concluded that the dynamic characteristics of neurons in electrical fields can be determined by neuronal morphology and neuronal adaptability [18]. Ha et al. investigated the influence of the Ca$^{2+}$-activated chloride channel anoctamin-2 on spike-frequency adaptation in thalamocortical and central nervous system neurons [19, 20]. Barraca et al. studied the influence of spike-frequency adaptation on the dynamics of a balanced network of neuronal models [13]. Chang et al. investigated the effects of external electric fields at extremely low frequencies on the adaptability of neurons in the Ermentrout model [21]. However, the influences of TMAS on the spike-frequency adaptation of neurons are still unknown. Thus, we applied TMAS to the Ermentrout model to analyze the effects of TMAS on neuronal spike-frequency adaptation.

The Ermentrout model is a typical neuron model that can be considered as a reduced one-compartment counterpart of the Traub–Miles model [22]. Both M-type current and AHP-current were introduced to the Ermentrout model during the neuronal spike-frequency adaptation simulations [17, 23, 24]. The membrane potentials of sensory neurons can be changed by external physical stimulation [16, 17]. Thus, when the Ermentrout model is stimulated by focused ultrasound waves in a static magnetic field, a stimulus current is added to the neuronal model, and a spike is emitted when the membrane potentials exceed the threshold. A spike-frequency curve is generated by assigning the inverse interspike interval from the membrane potential curve for each spike [25–28].

In our study, we extend the Ermentrout model by adding the electric current induced by TMAS to the membrane balance equation. We adopt an additional adaptation M-type current to study the firing characteristics and analyze the membrane potential curves as well as the spike-frequency curves as we vary the ultrasound and magnetic field parameters, such as magnetic flux density, ultrasonic intensity, fundamental ultrasonic frequency, modulation frequency, and duty cycles. We also discuss the changes in adapted onset spike-frequency curves under magneto-acoustical parameters based on different initial values of adaptation variables. We investigate the characteristics of spike-frequency adaptation for the Ermentrout neuron under TMAS through computational simulation and provide some discussion.

## Methods

### TMAS principle

**Fig. 1:**
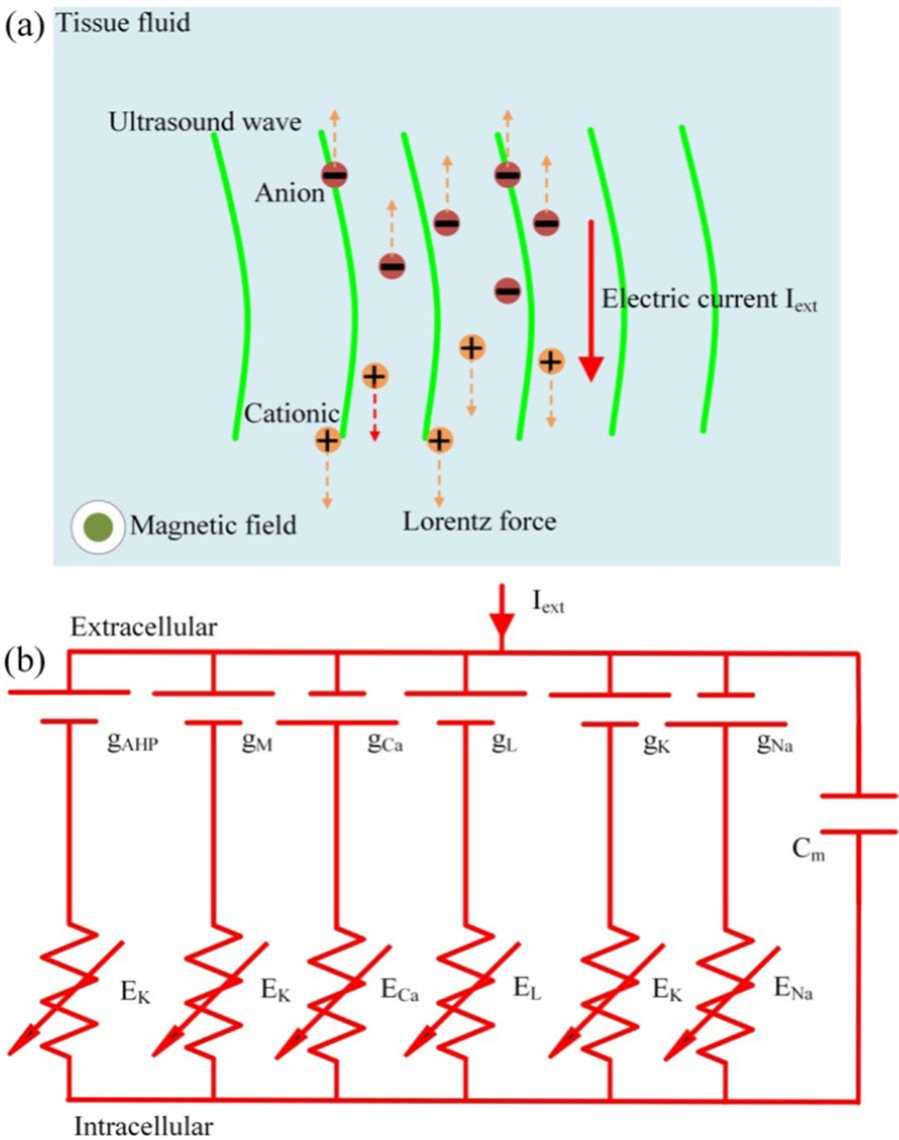
**a** Schematic of the TMAS principle; **b** Circuit diagram of the Ermentrout model under an electric current ($I_{ext}$)

A schematic showing the principle of TMAS is presented in Fig. 1a. In TMAS, a low-intensity focused ultrasound beam can drive the charged ions in tissue fluid. Combined with the static magnetic field, Lorentz forces in opposite directions generated on positive and negative charges. The moving charged ions forced by the Lorentz force form an electric current, $I_{ext}$, which is perpendicular to the direction of the static magnetic field and the ultrasound wave. In this study, we establish the Cartesian coordinate axes, in which ultrasound waves longitudinally propagate along the *z*-axis, the static magnetic field and the created current density are in the *x*-axis and the *y*-axis respectively. The relationship between the electric current density, $J_{y}$, and the ultrasound and magnetic parameters can be expressed as follows [29, 30]:

1 $$J_{y} = \sigma B_{x} \sqrt{ \frac{{2\Gamma}}{{\rho c_{0}}} }\sin ( 2\pi f_{u}t )$$

where $\sigma$ is the tissue conductivity, $B_{x}$ is the magnetic flux density, $\Gamma$ is the ultrasound intensity, $\rho$ is the tissue density, $c_{0}$ is the ultrasound speed, and $f_{u}$ is the fundamental ultrasonic frequency. This electric current density, $J_{y}$, can be represented as the external input current $I_{ext}$ of the Ermentrout neuron under TMAS.

In the simulation, to generate a sodium current and action potentials through stimulation, the fundamental ultrasound wave must be a sine wave with a DC offset [12]. The ultrasonic intensity influence the current density in nerve tissue. In the literature, both continuous and modulated ultrasonic waves are applied to stimulate the motor cortex, and both can safely and repeatedly evoke local field potentials [8]. Thus, we used pulsed ultrasound waves modulated by a square wave and continuous waves in our computational study. These waves obey the following equation:

2 $$x(t) = \begin{array}{*{20}{l}}{\left\{ {\begin{array}{*{20}{l}}{\left( {n - 1} \right)\frac{1}{{RF}} < t \le \left[ {\left( {n - 1} \right) + DC} \right]\frac{1}{{RF}},}\\{0,}\end{array}} \right.}\\{n = 1,2,3 \ldots }\\{others}\end{array}$$

where *RF* and *DC* are the modulation frequency and the duty cycle, respectively. Thus, the stimulus current, $I_{ext}$, generated by TMAS can be expressed as follows:

3 $${I_{ext}} = \begin{array}{*{20}{l}} {\left\{ {\begin{array}{*{20}{l}} {\sigma {B_x}\sqrt {\frac{{2\Gamma }}{{\rho {c_0}}}} \left( {\sin \left( {2\pi {f_u}t} \right) + 1} \right),}\\0 \end{array}} \right.}\\{\left( {n - 1} \right)\frac{1}{{RF}} < t \le \left[ {\left( {n - 1} \right) + DC} \right]\frac{1}{{RF}},n = 1,2,3 \ldots }\\{others}
\end{array}$$

The fixed parameters of this mathematical expression are listed in Table 1 [12].

**Table 1:** Fixed parameters for the electric current generated by TMAS

$\sigma$	0.5 Siemens/m	*DC*	10–95%
$B_{x}$	0.5–8 T	*RF*	1–100 Hz
$f_{u}$	200–700 kHz	$\rho$	1120 kg/m^3^
$\Gamma$	0.2–4 W/cm^2^	$c_{0}$	1540 m/s

### The Ermentrout model

The Ermentrout model is advantageous for describing voltage and current changes in the neuronal membrane. A circuit diagram of the Ermentrout model with external current, $I_{ext}$, is exhibited in Fig. 1b. The expression of the membrane potential can be described as follows [22]:

4 $$\begin{aligned} C_{m}\frac{dV}{dt}=-I_{Na}-I_{K}-I_{L}-I_{Ca}-I_{M}-I_{AHP}+I_{ext} \end{aligned}$$

where $C_{m}$ is the membrane capacity, $I_{ext}$ is the input stimulus current in tissue fluid, and *V* is the transmembrane voltage component generated by $I_{ext}$. The voltage-gated currents $I_{Na}$, $I_{K}$, and $I_{Ca}$ are given by the following equations:

5 $$\begin{aligned} &I_{Na}=g_{Na}m^{3}h\left( V-E_{Na}\right) \\&I_{K}=g_{K}n^{4}\left( V-E_{K}\right) \\&I_{Ca}=g_{Ca}\left( 1+\exp \left( -\left( V+25\right) /5\right) \right) ^{-1}\left( V-E_{Ca}\right) \end{aligned}$$

where $g_{Na}$ and $E_{Na}$ are the maximum conductance and the reversal potentials of sodium channels, respectively. Similarly, $g_{K}$, $E_{K}$, and $g_{Ca}$, $E_{Ca}$ represent the same parameters for the potassium and calcium channels, respectively. The kinetics of the gating variables $x\in m,n,h$ are given by

6 $$\begin{aligned} &\frac{dx}{dt}=\alpha _{x}\left( 1-x\right) -\beta _{x}x \\&\alpha _{m}=0.32\left( V+54\right) /\left( 1-\exp \left( -\left( V+54\right) /4\right) \right) \\&\beta _{m}=0.28\left( V+27\right) /\left( \exp \left( \left( V+27\right) /5\right) -1\right) \\&\alpha _{h}=0.128\exp \left( -\left( V+50\right) /18\right) \\&\beta _{h}=4/\left( 1+\exp \left( -\left( V+27\right) /5\right) \right) \\&\alpha _{n}=0.032\left( V+52\right) /\left( 1-\exp \left( -\left( V+52\right) /5\right) \right) \\&\beta _{n}=0.5\exp \left( -\left( V+57\right) /40\right) \end{aligned}$$

The leak current can be expressed as follows:

7 $$\begin{aligned} I_{L}=g_{L}\left( V-E_{L}\right) \end{aligned}$$

where $g_{L}$ and $E_{L}$ represent the maximum conductance and the reversal potential of the chlorine channel, respectively.

The M-type current is a kind of depolarization-activated and hyperpolarizing potassium current that decays with the activation of muscarinic acetylcholine receptors. It obeys the following expression:

8 $$\begin{aligned} I_{M}=g_{M}w\left( V-E_{K}\right) \end{aligned}$$

where $g_M$ is the maximum conductance, and *w* is the gating variable.

The AHP-type current is activated by the influx of calcium that results from firing and can be expressed as

9 $$\begin{aligned} I_{AHP}=g_{AHP}[Ca^{2+}]\left( 30+[Ca^{2+}]\right) ^{-1}\left( V-E_{K}\right) \end{aligned}$$

where $[Ca^{2+}]$ is the intracellular calcium concentration.

The kinetics of the M-type current *w* can be expressed as follows:

10 $$\begin{aligned} \tau _{w}\frac{dw}{dt}=\frac{1}{1+\exp \left( -\left( V+20\right) /5\right) } -w \end{aligned}$$

where $\tau$ is the time constant. The kinetics of $[Ca^{2+}]$ are:

11 $$\begin{aligned} \frac{d[Ca^{2+}]}{dt}=0.002I_{Ca}-0.0125[Ca^{2+}] \end{aligned}$$

The Ermentrout model is a reduced one-compartment counterpart of the Traub–Miles model. We introduce both M-type currents and AHP-type currents to establish the modified Ermentrout model for adaptation. However, when we simulated the neuronal spike-frequency adaptation, either an M-type current or an AHP-current was considered. In our simulation, we take the M-type current model as an example. Therefore, we set $I_{AHP}$ of the Ermentrout model constant in this paper. The fixed parameters used for the Ermentrout model in this study are listed in Table 2 [25].

The simulation was performed with MATLAB Simulink software (2018, MathWorks, USA). The main code used for the simulation has been deposited in a publicly available repository. It will be freely available to any scientist wishing to use them for non-commercial purposes.

**Table 2:** Fixed parameters for the Ermentrout model

$C_{m}$	1.0 $\upmu$F/cm$^{2}$	$E_{Na}$	+ 50 mV
$g_{Na}$	100 mS/cm$^{2}$	$E_{K}$	− 80 mV
$g_{K}$	80 mS/cm$^{2}$	$E_{L}$	− 67 mV
$g_{L}$	0.1 mS/cm$^{2}$	$E_{Ca}$	+ 120 mV
$g_{Ca}$	1 mS/cm$^{2}$	$g_{AHP}$	0 mS/cm$^{2}$
$g_{M}$	16 mS/cm$^{2}$	$\tau _{w}$	100 ms

### Adaptation of the Ermentrout model

Adaptation is a significant phenomenon of neurons in neural signal processing. The membrane potentials of sensory neurons can be changed by external physical stimulation. Therefore, we pay close attention to the membrane potential curve of the adaptive model under TMAS. A spike occurs whenever the membrane potential exceeds its upper threshold. We can obtain spike-frequency by assigning each time the inverse inter-spike interval that contains this time. Both the membrane potential curves and spike frequency curves exhibit the adaptation processes. In the spike-frequency curve, two frequencies need special attention. One is the initial spike-frequency, $f_{0}$, which denotes the inverse of the first interspike interval in the membrane potential curve; the other is the steady-state spike-frequency, $f_{\infty }$, which represents the stable spike-frequency after an extended period. In addition, the time from the initial to steady-state spike-frequency, which is the settling time, is of interest for spike-frequency adaptation.

Other adaptive characteristics of neurons can be well captured by the onset spike-frequency curve. The onset spike-frequency curve, $f_{0}\left( X\right)$, is obtained by mapping the corresponding initial firing frequency $f_{0}$ to each input parameter *X*. Besides, to reveal the onset responses of neurons to stimulus intensity changes, we build the adapted onset spike-frequency curve, $f_{0}\left( X, A_{0}\right)$, which consists of different onset spike-frequency curves under different initial stimulus inputs $A_{0}$. The adapted curves indicate how the neuron being in a stable and fixed adaptation state will initially respond to new stimuli. While each neuron has exactly one onset and one steady-state spike-frequency curve, it has for each adaptation varaible a different adapted spike-frequency curve.

## Results

### Membrane potential curves and spike-frequency curves under different TMAS input parameters

**Fig. 2:**
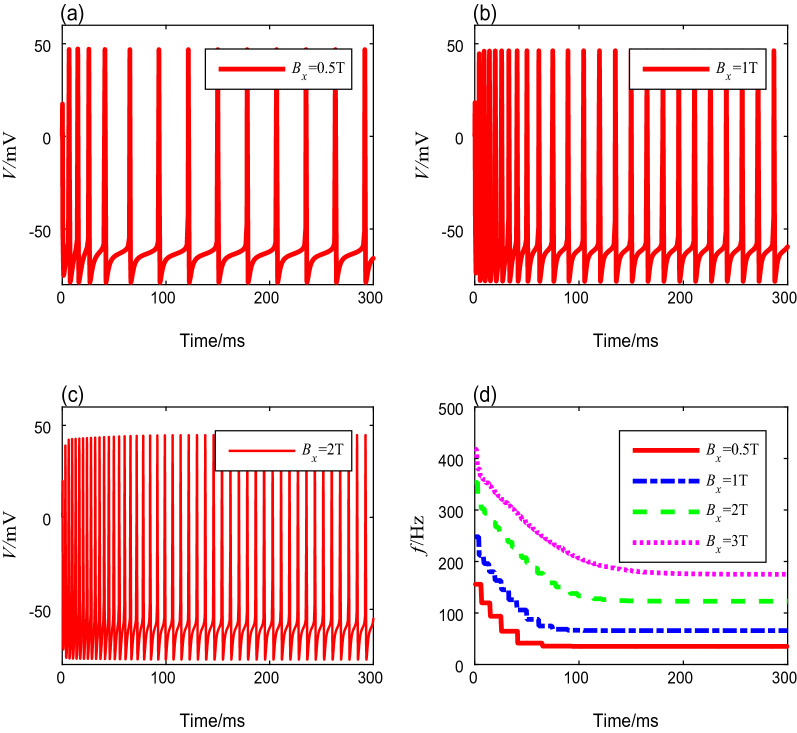
The membrane potential curves and spike-frequency curves under different magnetic flux densities

To investigate the effects of different TMAS input signals on the spike-frequency adaptation, we discuss the membrane potential curves and the spike-frequency curves under different TMAS input parameters. As described in the section of methods, five variable parameters ($B_x$, $\Gamma$, $f_{u}$, *RF*, and *DC*) of the modulated TMAS influence the spike frequency. To obtain the specific conclusions, we designate one as an adjustable parameter and fix the values of the others as follows: $B_{x}=2$ T, $\Gamma =3$ W/cm$^{2}$, $f_{u}=500$ kHz, $RF=1$ Hz and $DC=50\%$. Then, we discuss the characteristics of the membrane potential curves and the spike-frequency curves for different TMAS parameters.

i. To investigate the influence of TMAS on the neural spike frequency, the membrane potential is simulated based on the Ermentrout model using a range of magnetic flux densities from 0.5 to 8 T. The other TMAS input parameters are set to fixed values. Figure 2a–c show the plots of membrane potentials for magnetic flux densities of 0.5 T, 1 T, and 2 T, respectively. One can see that the spike-frequency ultimately settles into a steady state after a long period in which the intervals tend to be the same value. As shown in Fig. 2d, as $B_x$ increases, the spike-frequency curve shifts rightward and vertically toward those with a higher magnetic flux density, which means that the initial spike-frequency increases, and undergoes a longer transient process to attain the higher steady-state spike-frequency. In addition, the onset and steady-state spike frequencies of neurons, as well as the settling time, can be obtained through quantitative calculation, as shown in Table 3. We can conclude that as the magnetic flux density increases, both the onset and steady-state spike frequencies increase, and the transient process before the steady state requires more time.

**Table 3:** Spike frequencies and settling time with different magnetic flux densities

Magnetic flux density (T)	Onset spike frequency (Hz)	Steady-state spike freauency (Hz)	Settling time (ms)
0.5	155.9	35.3	65
1.0	248.0	65.7	103
2.0	353.4	122.8	123
3.0	418.1	175.2	201
ii To evaluate the influence of ultrasonic intensity on the neural spike-frequency adaptation under TMAS, different ultrasonic intensities from 0.2 to 4 W/cm^2^ were deployed in this simulation, while the other parameters are fixed values. The membrane potential curves corresponding to ultrasonic intensities of 0.5 W/cm^2^, 1 W/cm^2^, and 3 W/cm^2^ are shown in Fig. 3a–c. Similar to the results exhibited in Fig. 2, the spike-frequency decays with time and ultimately enters to a steady state. We also computed the spike frequencies, and the results are shown in Fig. 3d. We find that the spike-frequency curve shifts rightward and horizontally as the ultrasound intensity increases, which means that as ultrasonic intensity increases, the onset spike-frequency $f_{0}$ and the steady-state spike-frequency $f_{\infty }$ increase, and the settling time becomes longer. The detailed data are shown in Table 4.

**Fig. 3:**
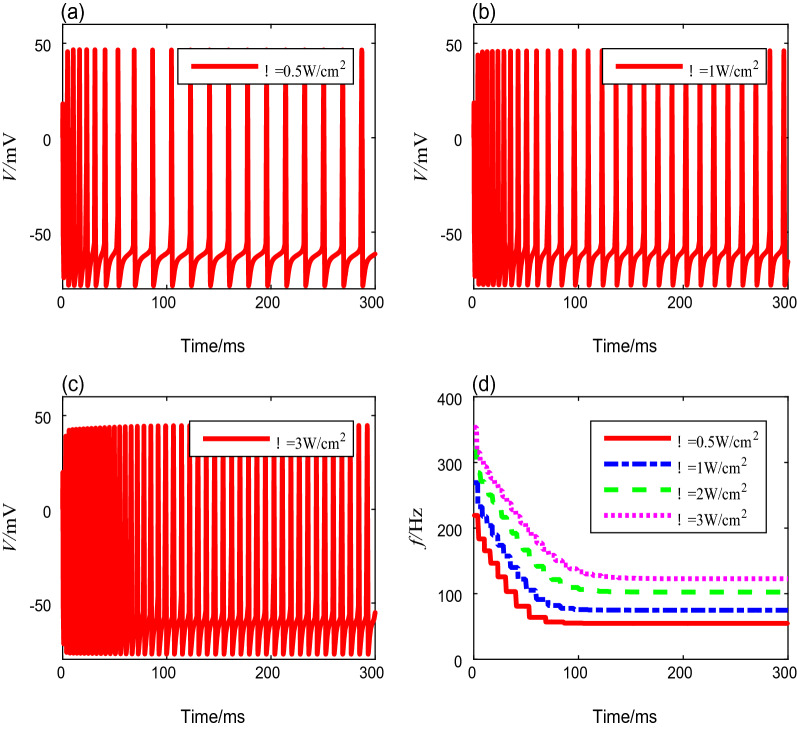
The membrane potential curves and spike-frequency curves with different ultrasonic intensities

**Table 4:** Spike frequencies and settling time with different ultrasound intensities

Ultrasound intensity (W/cm^2^)	Onset spike frequency (Hz)	Steady-state spike freauency (Hz)	Settling time (ms)
0.5	219.3	54.7	127
1.0	269.1	74.9	148
2.0	321.8	102.4	198
3.0	353.4	122.8	218
iii. Next, we analyze the neural spike-frequency adaptation under TMAS with various fundamental ultrasonic frequencies from 200 to 700 kHz. The other parameters are fixed to preset values. Figure 4a–c show the membrane potential curves corresponding to the ultrasound fundamental frequencies of 300 kHz, 400 kHz, and 600 kHz, respectively. No significant change occurs in the membrane potential of the adaptive model as the fundamental ultrasonic frequency increases. The quantitative calculation of the spike-frequency curves in Fig. 4d shows that the onset spike-frequency is always 353.4 Hz, and it reaches a steady-state value of 122.8 Hz after 218 ms under different fundamental ultrasonic frequencies. These results show that the fundamental ultrasonic frequency has no influence on the spike-frequency adaptation.

**Fig. 4:**
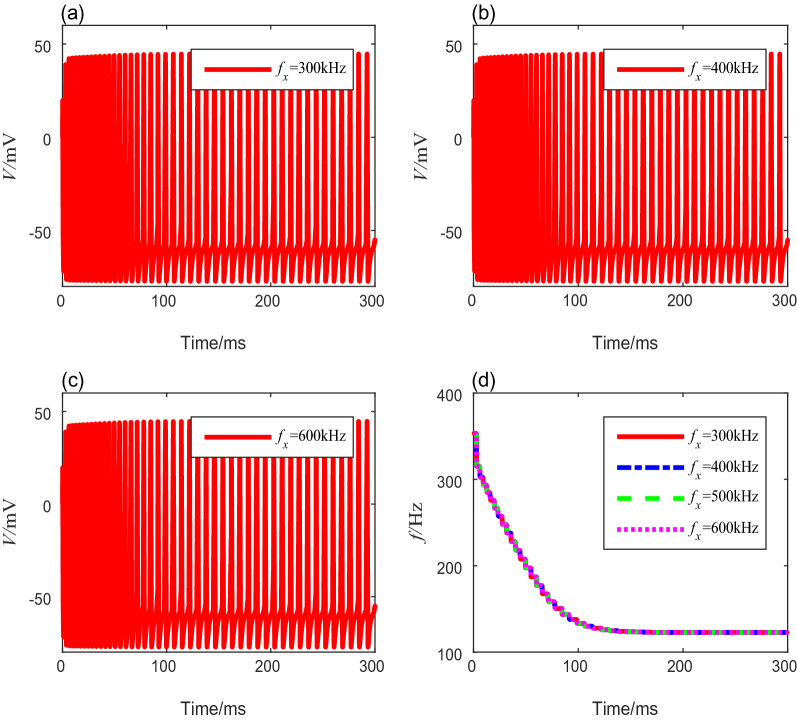
The membrane potential curves and spike-frequency curves under different ultrasound fundamental frequencies
iv Because the stimulus current $I_{ext}$ is generated by the modulated TMAS, the modulation frequency *RF* and the duty cycle *DC* have nonnegligible influences on the spike frequency. Different modulation frequencies from 1 to 100 Hz are deployed to the Ermentrout model to reveal the neuronal spike-frequency adaptation under the modulated TMAS. The values of duty cycles, ultrasonic intensity, magnetic flux density, and fundamental ultrasonic frequency are preset and fixed. The membrane potential curves corresponding to modulation frequencies of 20 Hz, 50 Hz, 80 Hz, and 100 Hz are shown in Fig. 5a–d, respectively. The spike frequencies settling time of neurons can be obtained through quantitative calculation, as shown in Table 5. Clearly, the interspike interval decreases as the modulation frequency *RF* increases. From Fig. 5e, f, we can see that the spike-frequency curves under different modulation frequencies have the same initial spike-frequency, and reach higher steady values more quickly with increasing modulation frequencies. However, when the modulation frequencies are too small, the spike frequency in both the transient and steady states fluctuates with time. In addition, the frequency of the spike-frequency curve increases as the modulation frequency increases, while the amplitude decreases.

**Fig. 5:**
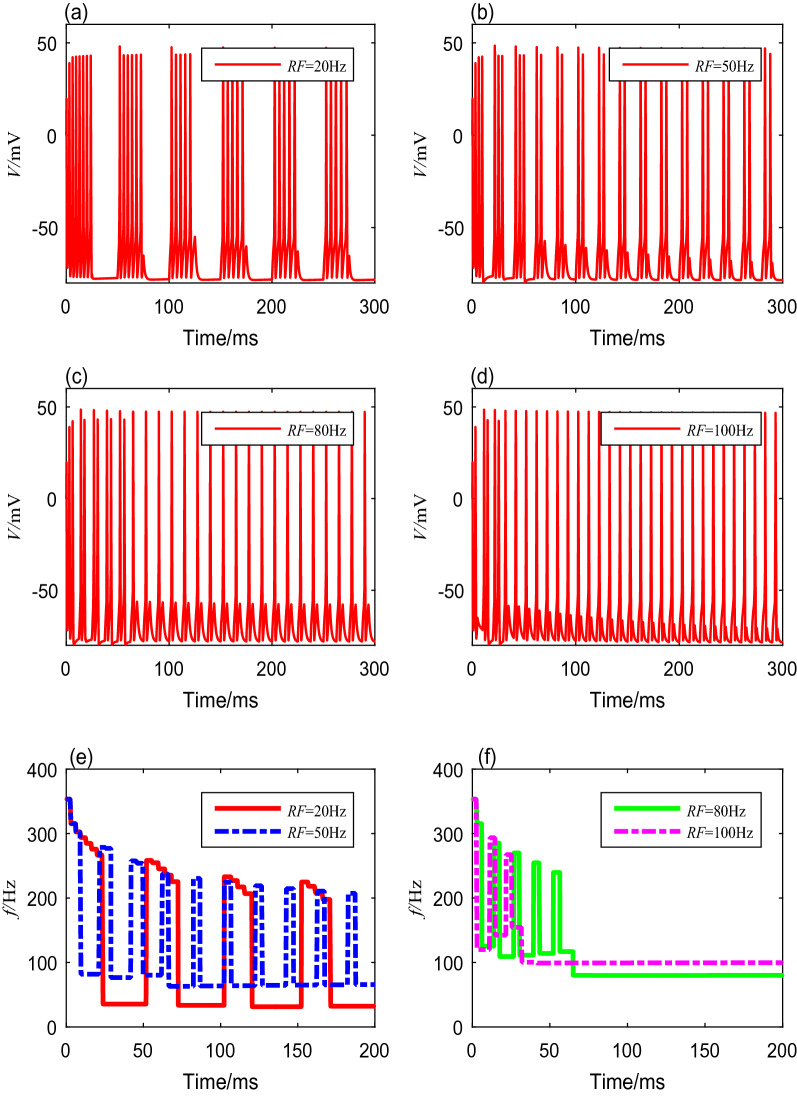
The membrane potential curves and spike-frequency curves with different modulation frequencies

**Table 5:** Spike frequencies and settling time with different modulation frequencies

Modulation frequency (Hz)	Firing pattern	Onset spike freauency (Hz)	Steady-state spike freauency (Hz)	Settling time (ms)
20	Bursting	353.4	32.6	$>200$
50	Bursting	353.4	66.7	$>200$
80	Spiking	353.4	80.0	66
100	Spiking	353.4	100	31
V. We also investigate the neuronal spike-frequency adaptation under TMAS using various duty cycles, from $10$ to $95\%$. We set the modulation frequency, ultrasonic intensity, magnetic flux density, and fundamental ultrasonic frequency using the fixed values mentioned above. The membrane potential curves with duty cycles of $30\%$, $50\%$, $70\%$, and $90\%$ are shown in Fig. 6a–d. The detailed spike frequencies and settling time with different duty cycles are shown in Table 6. The results indicate that a gap between a cluster decreases as the duty cycle increases. The membrane potentials produce bursting activities with the same initial spike frequency, but the spike frequency within each cluster decreases with both increasing time and increasing duty cycle, as shown in Fig. 6e, f.

**Fig. 6:**
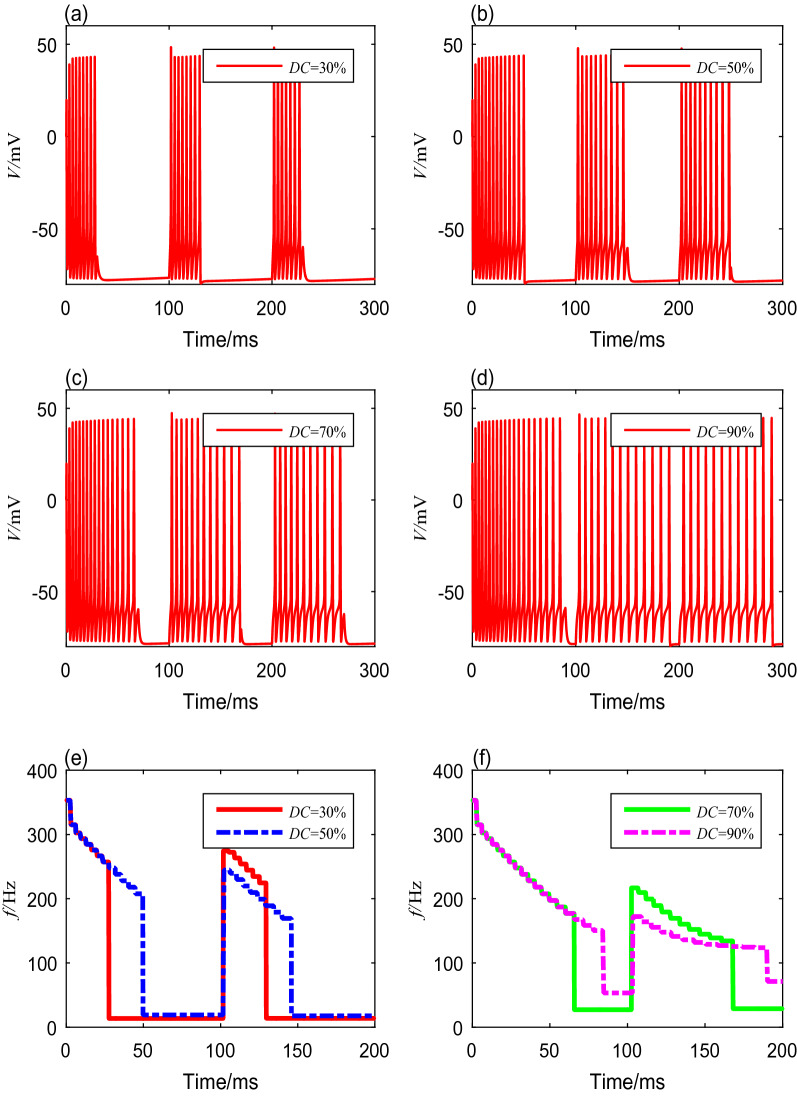
The membrane potential curves and spike-frequency curves under different duty cycles

**Table 6:** Spike frequencies and settling time with different duty cycles

Duty cycle (%)	Firing pattern	Onset spike freauency (Hz)	Minimal spike freauency (Hz)	Settling time (ms)
$30$	Bursting	353.4	241.7	$>200$
$50$	Bursting	353.4	185.6	$>200$
$70$	Bursting	353.4	139.0	$>200$
$90$	Bursting	353.4	132.2	179

#### Adapted spike-frequency curves with various initial stimulus inputs

**Fig. 7:**
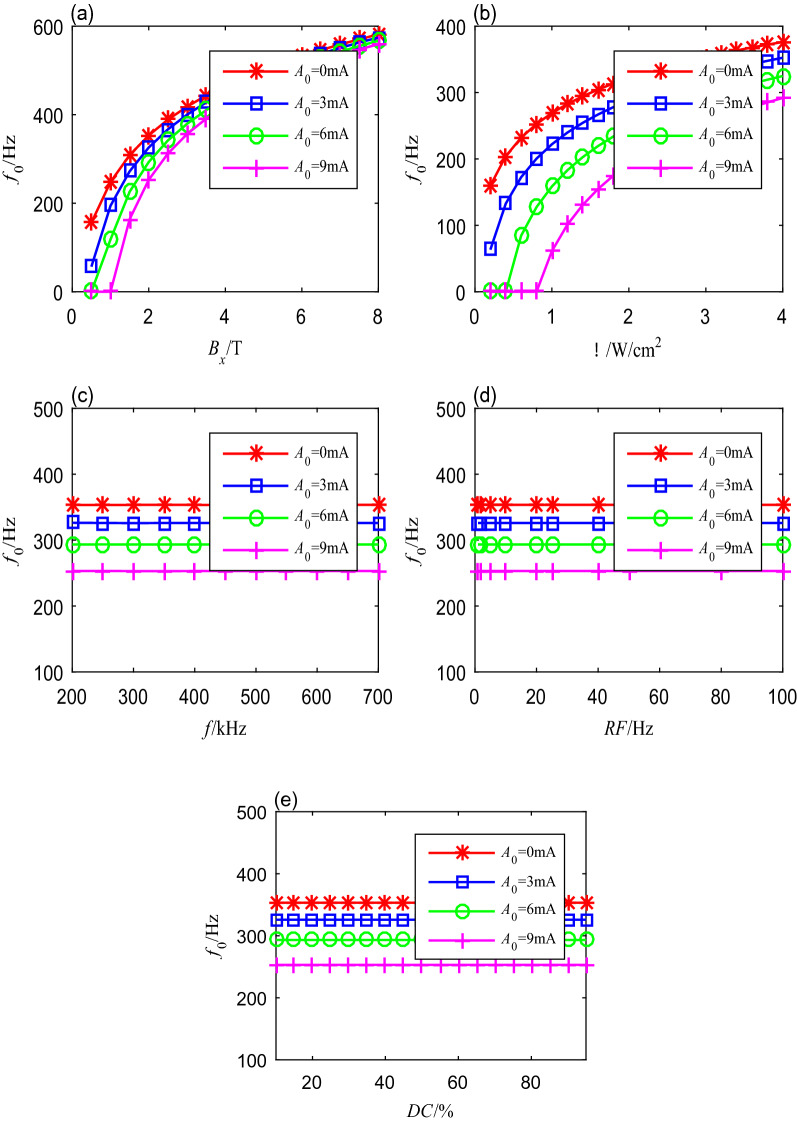
The adapted onset spike-frequency curves with different initial stimulus inputs

Adapted spike-frequency curves $f_{0}\left( X, A_{0}\right)$ represent the onset response of the neuron to input changes. They can be obtained by taking the different values of $A_{0}$, which represent the initial value of stimulus input. Figure 7a–e shows the adapted spike-frequency curves changing with the magnetic flux density, ultrasonic intensity, fundamental ultrasonic frequency, modulation frequency, and duty cycle, respectively. In these figures, the initial values of the stimulus input $A_{0}$ were equal to 0 mA, 3 mA, 6 mA, or 9 mA, respectively. Each adapting stimulus $A_{0}$ results in one adapted spike-frequency curve $f_{0}\left( X, A_{0}\right)$. Adapted spike-frequency curves indicate how the neuron being in a stable adaptation state will initially respond to new stimuli. All the curves together characterize the spike-frequency response of an adapting neuron.

The adapted spike-frequency curves under different magnetic flux densities and ultrasonic intensity are shown in Fig. 7a, b. When magnetic flux density varies from 0.5 to 4 T, and ultrasonic intensity varies from 0.2 to 2 W/cm^2^, the adapted spike-frequency curves have the same form, and the spike-frequency intervals distribute uniformly, which means the neuron has strong adaptation. However, as these two input parameters further increase, the adapted spike-frequency curves, $f_{0}\left( B_{x}, A_{0}\right)$ and $f_{0}\left( \Gamma , A_{0}\right)$, do not match the onset ones ($A_{0}$ = 0). We can conclude that the neuron has weak adaptation.

Other adapted spike-frequency curves under changing fundamental ultrasonic frequency, modulation frequency, and duty cycle are presented in Fig. 7c–e. We can observe that the initial spike frequencies do not change as the adaptation variables vary, whereas these onset spike frequencies decrease as the initial stimulation input increase. We can conclude that the fundamental ultrasonic frequency, modulation frequency, and duty cycle have no significant influence on the spike-frequency adaptation.

## Discussions

Based on the simulation results in the section of results, we analyzed the influence of different TMAS parameters on the adaptation process of the Ermentrout neuron. The conclusions demonstrate that both the initial and steady-state spike frequencies of the neuron increase with the increase of magnetic flux density or ultrasonic intensity of TMAS. According to the physiological mechanism of TMAS, the amplitude of the stimulus input increases as the magnetic flux density and ultrasound intensity increase, thus shortening the time required for membrane potential to reach the threshold. Therefore, both the initial spike frequency and the steady-state spike frequency of the neuron increase. The fundamental ultrasonic frequency of TMAS is in kilohertz level. Such a high-frequency transformation of the input intensity has little influence on the current in the tissue fluid of a neuron. Therefore, changes in the fundamental ultrasonic frequency of TMAS do not affect the neural spike frequency. Besides, we used a square wave to modulate the sinusoidal ultrasonic wave with offset in our simulation. Changes in the modulation frequency and duty cycle alter the current waveform of TMAS and then affect the frequency of the stimulus input acted on neurons. Therefore, changes in the modulation frequency and duty cycle lead to changes in the firing mode and the firing rhythm of the neurons.

We analyze the influence of different TMAS parameters on the spike-frequency adaptation of the Ermentrout neuron based on the adapted spike-frequency curves with various initial stimulus inputs in the section of results. The results show that the fundamental ultrasonic frequency, modulation frequency, and duty cycle do not affect the neural spike-frequency adaptation. The adaptation state usually alters with the slowly changing stimulus input and is subtracted from the stimulus input, so that the average value of the stimulus input changes. As a result, only rapidly fluctuating stimulus signals can be transmitted. In other words, the higher the alternating frequency, the strong adaptation the neuron exhibits. TMAS used high-frequency ultrasound to act on neurons. Therefore, there is no effect on the neural spike frequency adaptation of the fundamental ultrasonic frequency, modulation frequency, or duty cycle. However, the increase of magnetic flux density and ultrasonic intensity directly causes the enhancement of the stimulus strength, and the adaptation current strongly fluctuates between spikes. Therefore, the stronger the TMAS input, the worse the adaptation exhibits. According to Kirchhoff’s current theorem, the dynamic equation of membrane potential is derived from the vector sum of ionic currents. Therefore, with the increase of adaptation current, the onset spike frequency of neurons decreased.

Adaptation, which represents not only the current state of adaptation but also the process of dynamic change, plays a significant role in neural information processing. In particular, a variety of firing currents lead to spike-frequency adaptation and can explain many phenomena, such as weak stimulation and enhanced rapid response to stimulation. Therefore, the adaptation current can be combined with TMAS and neurons to study the influence of TMAS on the adaptability of the neural network. This knowledge can help in exploring how TMAS regulates the biological nervous system.

## Conclusions

In this paper, we investigated how TMAS influences neuronal spike-frequency adaptation by exploring different ultrasonic and magnetic field parameters, and furthermore, how it affects neural information transmission. Based on a numerical analysis, the magnetic flux density and ultrasonic intensity are found to be the determining parameters of spike-frequency adaptation. In addition, while the different modulation frequencies and duty cycles may lead to different membrane potentials and spike frequencies, they have no influence on spike-frequency adaptation. Based on the numerical analysis of neuron model, this paper explores the adaptation of neurons to the TMAS input and the effect of the stimulus current on the discharge activity of biological nervous system. These results enrich the theoretical basis of TMAS as a potential clinical treatment of nervous system diseases.

## Data Availability

All data generated or analyzed during this study are included in this published article. The main simulation code that support the findings of this study are available in ’Github’ with the identifier [https://zenodo.org/badge/latestdoi/428488703].

## Abbreviations

TMAS: Transcranial magneto-acoustical stimulation
TFUS: Transcranial focused ultrasound stimulation
M-type currents: Voltage-sensitive potassium currents
AHP-type currents: Calcium-sensitive afterhyperpolarization currents
